# Characterisation of the Faecal Microbiome of Alpacas Raised in South Eastern Australia

**DOI:** 10.3390/ani15121748

**Published:** 2025-06-13

**Authors:** Imogen Boughey, Francisca Samsing, Evelyn Hall, Rachael Rodney, Russell Bush

**Affiliations:** 1 Sydney School of Veterinary Science, The University of Sydney, 425 Werombi Road, Camden, NSW 2567, Australia; 2 Fenner School of Environment & Society, The Australian National University, Building 141, Linnaeus Way, Canberra, ACT 2601, Australia; rachael.rodney@anu.edu.au

**Keywords:** alpaca, camelid, faecal microbiome

## Abstract

There is limited investigation of the alpaca microbiome on a global scale, with no previous research conducted in Australia characterising the faecal microbiome. The microbiome composition in other ruminants has been shown to impact feed efficiency, average daily gain and methane production. This study aimed to characterise the faecal microbiome of alpacas raised in south-eastern Australia and identify variation across geographic regions. Faecal samples were collected from 59 healthy adult female alpacas, aged between 15 months and 17 years in NSW, Australia. Firmicutes were identified as the dominant phyla, accounting for 57.78% of the cumulative abundance, followed by Bacteroidota (29.12%). These two phyla accounted for 90% of the taxa, with the cumulative abundance of Firmicutes and Bacteriodota significantly differing (*p* < 0.05) across locations. There was no effect of age on the frequency of microbes identified at either phyla or class levels. The alpaca’s production status only significantly affected the abundance of Firmicutes *Clostridia Oscillospirales* (*p* = 0.0026). The breakdown of the alpaca faecal microbiome identified here is consistent with previous ruminant and camelid studies. This study provides a valuable baseline for the microbiome characterisation of alpacas in south-eastern Australia and can be used as a baseline for further microbiome studies.

## Simple Summary

There is a large health and production knowledge gap for alpacas raised in Australia and further abroad with regard to their microbiome. This study aimed to characterise the faecal microbiome of alpacas raised in south-eastern Australia and identify variations across geographic regions. Faecal samples were collected from 59 healthy adult female alpacas, aged between 15 months and 17 years in NSW, Australia. Firmicutes were identified as the dominant phyla, followed by Bacteroidota. These two phyla accounted for 90% of the taxa, with the cumulative abundance of Firmicutes and Bacteriodota significantly differing across locations (*p* < 0.05). Age did not have any effect on the frequency of microbes identified at either phyla or class levels. The alpaca’s production status only significantly affected the abundance of Firmicutes *Clostridia Oscillospirales* (*p* = 0.0026). The characterisation of the alpaca faecal microbiome identified here is consistent with previous ruminant and camelid studies. This study provides a valuable baseline for the microbiome characterisation of alpacas in south-eastern Australia and can be used as a reference point for further microbiome studies.

## 1. Introduction

The ruminant microbiome plays an essential role in the absorption of nutrients and the feed efficiency [1,2]. In ruminants, the microbiome composition has been seen to impact weight gain and feed efficiency due to the role of key taxa in carbohydrate metabolism [3,4]. Variation from a healthy microbiome is linked to metabolic diseases, with an understanding of the role of taxa in the microbiome informing management practices and optimising animal health and growth [4,5]. As methane production from agriculture contributes to greenhouse gas emissions, understanding the composition of the ruminant microbiome and factors that influence metabolic function has become a focus, specifically the functional and compositional microbiome [5,6,7].

Alpacas are pseudo-ruminants, with 3 compartments in their foregut, relying on gut microbial communities to break down plant cell wall carbohydrates, similar to true ruminants [8]. Microbiome compositions in ruminant species are highly variable and can be impacted by a variety of factors, including diet composition and husbandry practices [2,9,10]. Understanding the alpaca microbiome is important for improving management practices for optimal alpaca health and production. The proportion of Firmicutes and Bacteroidota as the dominant phyla in ruminants influences feed efficiency and average daily gain [4]. As pseudo-ruminants, the limited research on the alpaca microbiome globally has indicated similarities with the dominant microbiome of true ruminants. American-based studies determined that Firmicutes and Bacteroidetes are the dominant phyla found in the faecal and gut microbiome of alpacas and camels [8,9,10,11]. However, there is variation in the dominant taxa, as species in the Eubacterium phyla have also been highlighted in both the USA and China [12,13]. The variation in findings across different geographical locations highlights the need to investigate environment-specific microbiome communities in alpacas. Understanding the composition of the microbial communities in alpacas presents an opportunity to improve nutritional management and gastrointestinal care to benefit the animals and production system. Understanding the microbiome of ruminants has had increased interest recently due to the role of methanogenic archaea in the production of methane in the rumen [6]. Alpacas have been identified to have lower methane emissions compared to sheep raised in the same environments due to the differences in the microbiome [12]. Identifying these changes across different locations and conditions may provide insight for future management of other ruminant species.

The microbiome has traditionally been difficult to investigate as it requires specialist expertise to collect and process rumen samples. Studies such as [11] have started to characterise the microbiome using faecal samples, providing a non-invasive option for analysing microbiome communities. The faecal microbiome in ruminants is comparable to the microbial community found in the lower digestive tract [14,15]. To our knowledge, no studies have been conducted that examine the faecal microbiome of alpacas in Australia, and there are varying microbial populations reported between the USA and China. This study aimed to characterise the faecal microbiome of alpacas raised on the east coast of Australia to create a reference base for future research and veterinary applications.

## 2. Materials and Methods

### 2.1. Faecal Collections

**Figure 1:**
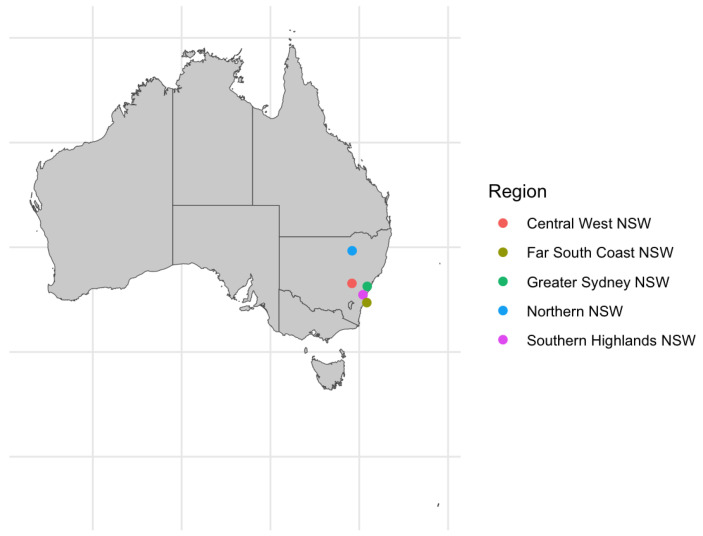
Distribution of alpaca faecal sample collection locations in Australia.

Faecal samples were collected from healthy, breeding female alpacas aged between 15 months and 17 years from 5 farms across New South Wales, Australia, in September 2023. The farms were located in the Southern Highlands, Far South Coast, Central West, Greater Sydney and New England regions (Figure 1). The locations were selected as representative distribution of Australian alpaca farms based on survey data [16]. Table 1 oulines the primary pasture species and supplement feed on offer at each location. All alpacas were female and categorised into one of the following production status categories: Empty (not pregnant), Empty with Cria at Foot (not pregnant and lactating), Pregnant, and Pregnant with Cria at Foot (pregnant and lactating). A total of 60 samples were collected, 12 from each location. Table 2 presents data on the individual alpacas. Faecal samples were collected from animals restrained in a purpose-built race using a lubricated glove to scoop faecal pellets from the rectum. Faeces were collected in accordance with procedures approved by the University of Sydney Animal Ethics Committee (Project Number 2023/2333).

### 2.2. DNA Extraction and Bioinformatics

Samples were frozen at −20 °C on the day of collection and until undergoing freeze drying for 72 h. Freeze-dried samples were stored at −20 °C until DNA extraction. DNA was extracted using the QIAamp Powerfaecal Kit (Qiagen, Hilder, Germany) per the manufacturer’s instructions. PCR products were further processed at the Ramaciotti Centre for Genomics (UNSW, Sydney, Australia). Illumina MiSeq 2 × 300 bp (Illumina, San Diago, CA, USA) sequencing was used to amplify the 16S rRNA, V3–V4 regions using the forward primer ‘CCTACGGGNGGCWGCAG’ and reverse primer ‘GACTACHVGGGTATCTAATCC’. Quality control was >70% sequences above Q30. Microbiome bioinformatics analysis was performed with QIIME2 2024.10 [17]. Primers were removed with cutapdat, trimming was conducted with the quality filter q-score [18] and denoising was completed with DADA3 [19] (via dada2 denoise-paired). One sample was removed during filtering due to a frequency below 2000. Taxonomy was assigned using classify-sklearn [20], naïve Bayesian classifier [21] and the SILVA NR 99 database. OTUs (operational taxonomic units) classified as mitochondria, Eukaryota and chloroplast were filtered before analysis. Abundance calculations and the bacterial community structure were assessed using the Bray–Curtis distance calculation with a principal coordinate analysis plot using R [22]. The original data presented in the study are openly available in the Sequence Read Archive (SRA) at PRJNA1264719.

### 2.3. Statistical Analysis

Analyses were performed using R Version 4.4.1 (R Core Team 2024) and Microsoft Excel 2018 [23]. Linear models were used to determine the effect of the location, production status and age on the presence of the microbial communities identified in the bioinformatics steps down to an order level. Linear modelling was conducted on the top OTUs, which included the populations contributing up to 90% or greater of the cumulative abundance when sequencing results were pooled. A *p*-value of <0.05 was considered significant.

## 3. Results

### 3.1. Faecal Microbiome Population

The average number of 16S rRNA gene sequences per sample was 24,082.27 (±9572.91). Firmicutes were the dominant phyla identified, accounting for 55.35% of the taxa in the pooled samples, followed by Bacteroidota (31.30%) (Table 3). A detailed breakdown of the taxa found at phyla, class and order levels that account for the top OTUs is presented in Table 3.

### 3.2. Effect of Location, Age and Production Status

**Figure 2:**
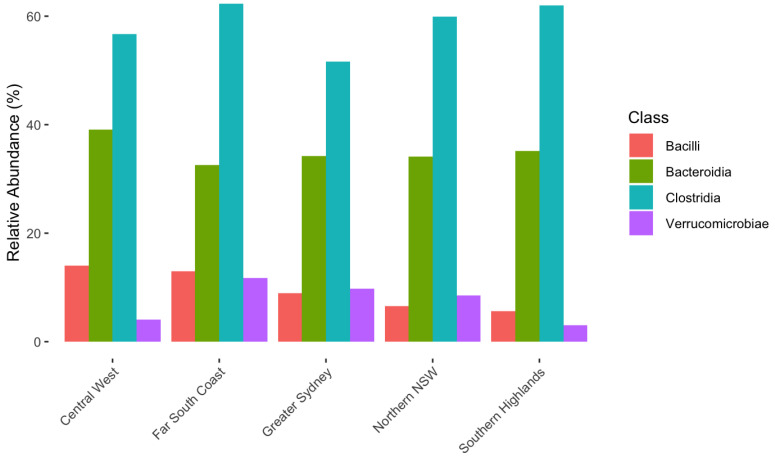
Variation of relative abundance of class OTUs making up top 90% by region in alpaca faecal microbiome.

**Figure 3:**
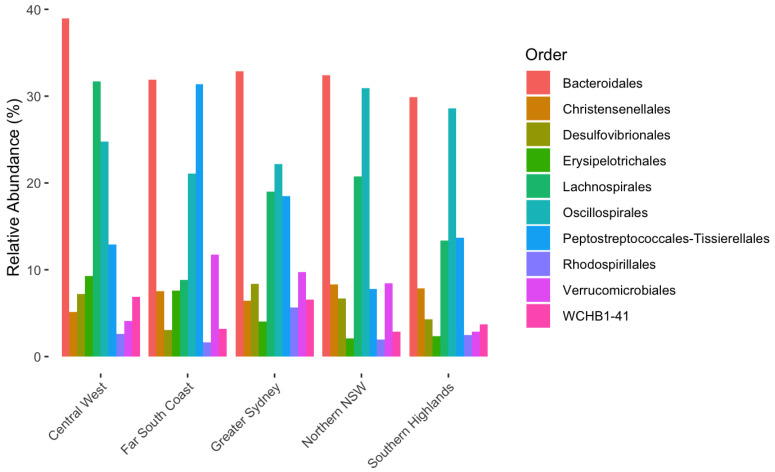
Variation of relative abundance of order OTUs making up top 90% by region in alpaca faecal microbiome.

All the top OTUs at a phylum, class and order level were significantly affected by region (*p* < 0.05). The phylum Firmicutes accounted for between 53.9% and 61.5% of the alpaca biome, whilst Bacterioda contributed between 27.3% and 33.7%. The distribution of the top taxa for Class and Order levels can be seen in Figure 2 and Figure 3.

**Figure 4:**
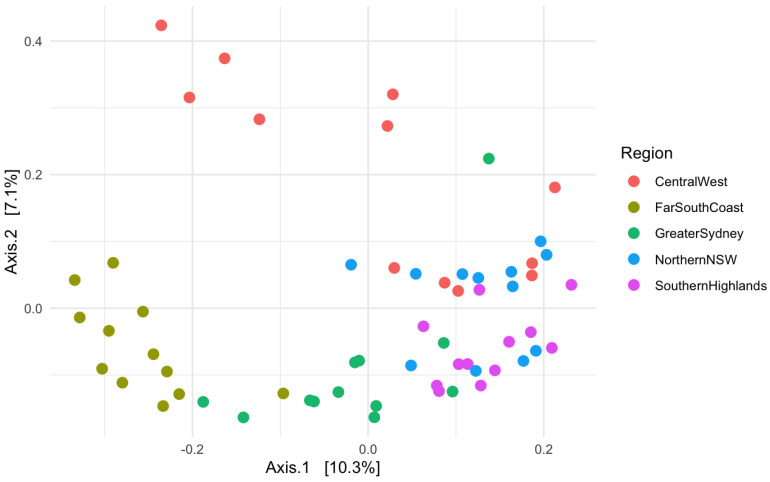
Principal coordinate analysis showing the analysis of faecal microbiome in alpacas across different regions of NSW.

The principal coordinate analysis calculated using the Bray–Curtis distance accounted for 17.4% of the variation between groups. The limited clustering indicates that Northern NSW, Southern Highlands and, to a lesser degree, the Central West regions were more similar compared to the Far South Coast and Greater Sydney (Figure 4).

At an order level, Firmicutes Clostridia Oscillospirales (*p* = 0.0026) was the only taxa significantly impacted by status with Empty animals displaying a significantly higher abundance of Firmicutes Clostridia compared to animals that were Pregnant and Pregnant with Cria at Foot. No other taxa displayed significance. There was no effect of age displayed at the phyla, class or order levels (*p* > 0.05).

### 3.3. Methanogens Present in Alpaca Faecal Microbiome

The Archaea Euryarchaeota phyla accounted for 0.17% of the taxa in the alpacas studied. At a class level, from the Euryarchaeota phylum, *Methanobacteriales* accounted for 0.18% of the total taxa, *Methanomassiliicoccales* 0.09% and *Methanomicrobiales* 0.01%.

## 4. Discussion

There is a paucity of information on the microbial community in alpacas with a small number of studies globally, mainly in the USA and China, exploring alpaca microbiome composition and diversity. In other species, including cattle, changes in taxa have been associated with desired improvement in health and production such as higher weight gain during weaning. The dominant taxa now identified in Australian alpacas display similar ratios when compared to other ruminants. Future research building upon this characterisation of the alpaca microbiome in Australia will provide a new pathway for veterinary management of alpacas in small-scale and commercial production systems.

The majority of alpaca microbiome studies have sampled forestomach fluid and digesta from other areas along the gastrointestinal tract with only three studies using faecal samples for analysis. Of these, ref. [24,25] focus on isolating novel species of Bacteroides and Clostridium, respectively, whilst [11] looked at the association between the microbiome and endoparasitism making it difficult to meaningfully compare this characterisation with these studies. Although prior use of faecal samples to analyse alpaca microbiome had different focal points, the results support Firmicutes and Bacteroidota being the dominant two taxa in alpacas despite different geographical locations [9,11,24,25]. Firmicutes and Bacteroidota have been widely reported as dominant taxa in other common ruminant species, including cattle, sheep and goats [4,26,27,28,29]. The proportion of these two groups of taxa varies, with Firmicutes consistently displaying a higher proportion than Bacteriodota, aligning with the results in this study [4,26,28]. The faecal microbiome of camels has been studied more widely compared to alpacas exhibiting similar results to the microbial composition of this study, despite different locations and environmental conditions. Firmicutes (59.7%), Bacteroidota (24.3%), as well as Verrucomicrobiota (8.09%), have been reported as the three dominant taxa in adult Bacterian camels raised in Mongolia, with similar levels of relative frequency as reported here [30]. The similarity across the different environments indicates that at a phlya level, the Firmicutes and Bacteroidota are consistently the dominant taxa [9,11,24,25]. This study also indicates that the environment can impact the proportion of both phyla present in the faecal samples. This trend was also reported in India in camels where the faecal microbiome was compared between two different management and feeding systems [10]. Conversely, that study found that in an extensive system most similar to Australia, Proteobacteria was the most abundant phyla followed by Firmicutes, then a smaller proportion of Bacteroidota, highlighting a potential difference in camelid species that requires further research [3,30]. The variation among locations needs to be researched further to investigate the impact of environmental factors due to location or the contribution of management practices on microbiome composition.

Pregnancy and lactation have various effects on the physiology of female ruminants, including altering metabolic rate and nutritional requirements along with the composition of the rumen microbiome fluid [31,32,33]. Lima at al. [31] found that the structure of the microbial communities present in the rumen fluid of dairy cows changed between prepartum and 1 week postpartum (lactation) and that certain bacterial taxa were associated with differences in milk production and composition. The abundance of Firmicutes in Hu sheep has been found to be higher during pregnancy and lactation whilst the abundance of Bacteroidota was lower when compared to non-pregnant (empty) sheep [32]. However, this trend is not consistent with breed, as Suffolk sheep in the same study showed higher levels of Bacteroidota taxa during pregnancy and lactation [32]. Although the taxa reported vary between studies in different ruminant species (cattle, sheep and goats), differences in the microbiome are consistently identified between pregnant, lactating and empty animals [32,33,34]. Further understanding the impact of variation at different production status’ could provide opportunities to improve management recommendations and health using faecal samples as a minimally invasive sample collection method. Furthermore, studies conducted in camels have identified different key taxa in animals at 2 months of age vs. adult animals between 1 and 3 years [30]. This variation has been connected to the changes in diet and/or physiology as alpacas/camels age, which in turn influence microbial communities and their role in digestion and immune system development [30]. However, in this study, only adult female alpacas were examined, and age did not have any impact. This suggests a need for future studies to compare alpacas of different ages.

Alpacas have been reported to produce less methane compared to other domesticated ruminants such as sheep and cattle [12,35]. The Archaea *Methanbrevibacter* clade has been identified as the key methanogen from the Euryarchaeota phyla responsible for ruminant methane production [6,36]. Both *Methanbrevibacter* and *Methanosphaera* have previously been identified as methanogens present in the forestomach of alpacas, with *Methanbrevibacter* as the dominant methanogen [13], which is consistent with the results of this study using faecal samples. In this study, the Euryarchaeota taxa only accounted for 0.17% of phyla, with *Methanbrevibacter* the dominant taxa in this phyla, which is less than what has been reported in sheep [12] and other ruminants [35]. Pei et al. [12] reported that methanogens accounted for 1.38% of the microbial forestomach community in alpacas, which was lower than the 1.92% they reported in sheep, following a similar trend reported by [37]. This variation in the proportion of methanogens identified between these studies could be attributed to the difference in location and management conditions and requires a more detailed investigation. The lower percentage of methanogens, alongside nutrition and other physiological differences in the gastro-intestinal tract has been postulated as the reason for the reduced methane production in alpacas compared to other ruminants [35,37,38].

The practical applications of understanding the alpaca microbiome at a global and local scale are important in improving the overall knowledge base of alpaca health and management in their context as a farmed production animal. The impact of endoparasites on the faecal microbiome has been investigated with endoparasite presence linked to a small proportion of variation between microbial composition and stronglyid egg populations [11]. Firmicutes have been identified to breakdown carbohydrates into energy [3], with the Firmicutes-to-Bacteroidota proportion impacting feed efficiency in ruminants and average daily gain (ADG) in cattle [3,4]. Maslen et al. [4] reported that in weaning cattle, the individuals with a lower production efficiency (reduced ADG) had a lower frequency of Firmicutes and higher Bacteroidota compared to those with a higher efficiency, highlighting that understanding the microbiome communities is important in managing ruminant nutrition and production efficiency.

The similarity of microbiome key taxa at a phylum level across common ruminant species in agriculture has been acknowledged, supporting the theory of a core rumen microbiome [3]. The similar key taxa among sheep, cattle and alpacas, across a variety of locations suggests that other ruminant models such as sheep, cattle or camels may be useful for basic knowledge in alpacas; however, as there is variation in physiology, more species-specific models and research needs to be conducted to improve alpaca welfare, veterinary care and management practices.

## 5. Conclusions

The faecal microbiome of alpacas in Australia is consistent with ruminant and camelid studies from across the globe. Firmicutes and Bacteroidota were the key phyla identified by this study and align with other ruminants including cattle, providing a baseline for further research into the production implications of changes in microbiome composition. The results of this study support the theory of a core rumen microbiome at a phylum level, indicating that other ruminant models may be the suitable starting point for alpaca-based care. However, species-specific knowledge still needs to be established.

## Figures and Tables

**Table 1:** Primary pasture species and supplementary feed.

Region	Primary Pasture Species	Supplemental Feed
Central West	Phalaris (*Phalaris aquatica*), Cocksfoot (*Dactylis glomerata*), Wallaby Grass (*Rytidosperma* spp), Microlena (*Microlena stipoides*), Tussock Grass (*Poa labillardierei*), White Clover (*Trifolium repens*), Subterranean Clover (*Trifolium subterraneum*), Perennial Ryegrass (*Lolium perenne*)	
Far South Coast	Kikuyu (*Cenchrus clandestinus*), Phalaris (*Phalaris aquatica*), Cocksfoot (*Dactylis glomerata*), Wallaby Grass (*Rytidosperma* spp.), Microlena (*Microlena stipoides*), Tussock Grass (*Poa labillardierei*)	Ad Lib Access to Hay Mix: Lucerne (Alfalfa) Hay (*Medicago sativa*), Oaten Hay, Clover Hay
Greater Sydney	Kikuyu (*Cenchrus clandestinus*), Microlena (*Microlena stipoides*), Couch (Cynodon dactylon)	Ad Lib Access to Hay Mix: Lucerne Alfalfa) Hay (*Medicago sativa*), Oaten Chaff, Horse Pellet Mix
Northern NSW	Phalaris (*Phalaris aquatica*), Cocksfoot (*Dactylis glomerata*), White Clover (Trifolium repens), Perennial Ryegrass (*Lolium perenne*)	
Southern Highlands	Kikuyu (*Cenchrus clandestinus*), Perennial Ryegrass (*Lolium perenne*)	

**Table 2:** Metadata for sample collection including basic husbandry practices.

Region	Age (Months)	N	Production Status	N	Vaccination	Drench	Recent Supplements
Far South	13–18	0	Empty with Cria	1	5in1	STARTECH	ADE and
Coast	19–29	1	Pregnant	9			Phosphorus
	30–47	2	Empty	2			Injection
	48+	9	Pregnant with Cria	0			
Central	13–18	0	Empty with Cria	2	5in1	NA	Selenium
West	19–29	1	Pregnant	0			1 mL
	30–47	2	Empty	9			
	48+	9	Pregnant with Cria	1			
Greater	13–18	0	Empty with Cria	2	5in1	AVERMEC	
Sydney	19–29	3	Pregnant	5		Dual	
	30–47	0	Empty	5			
	48+	9	Pregnant with Cria	0			
Southern	13–18	0	Empty with Cria	0	5in1	Startech	ADE
High-	19–29	0	Pregnant	1			Injection
lands	30–47	2	Empty	11			
	48+	10	Pregnant with Cria	0			
Northern	13–18	2	Empty with Cria	1	5in1	Ivermectin	
NSW	19–29	2	Pregnant	5		Injectable	
	30–47	0	Empty	6		(Cattle)	
	48+	8	Pregnant with Cria	0			

NA-not applicable.

**Table 3:** Relative abundance of top OTUs in alpaca faecal microbiome.

Phylum	
Firmicutes	57.78%
Bacteroidota	29.12%
Class	
Firmicutes Clostridia	51.76%
Bacteroidota Bacteroidia	29.16%
Firmicutes Bacilli	5.48%
Verrucomicrobiota Verrucomicrobiae	2.90%
Order	
Bacteroidota Bacteroidia Bacteroidales	29.05%
Firmicutes Clostridia Lachnospirales	21.41%
Firmicutes Clostridia Peptostreptococcales-Tissierellales	11.99%
Firmicutes Clostridia Oscillospirales	10.19%
Verrucomicrobiota Verrucomicrobiae Verrucomicrobiales	5.12%
Firmicutes Bacilli Erysipelotrichales	2.89%
Desulfobacterota Desulfovibrionia Desulfovibrionales	2.71%
Firmicutes Clostridia Christensenellales	2.40%
Verrucomicrobiota Kiritimatiellae WCHB1-41	2.00%
Proteobacteria Alphaproteobacteria Rhodospirillales	1.29%

## Data Availability

The raw sequencing data for this genome is publicly available in the SRA(PRJNA1264719).
